# A new estimation equation to assess body composition in an athletic population

**DOI:** 10.1080/15502783.2025.2504578

**Published:** 2025-05-28

**Authors:** Meghan K. Magee, Jennifer B. Fields, Angela Miller, Andrew R. Jagim, Debra Stroiney, Brittanie Lockard, Margaret T. Jones

**Affiliations:** a George Mason University, Patriot Performance Laboratory, Frank Pettrone Center for Sports Performance, Fairfax, VA, USA; b Kent State University, Exercise Science and Exercise Physiology, Kent, OH, USA; c University of Connecticut, Nutritional Sciences, Storrs, CT, USA; d George Mason University, School of Education, Fairfax, VA, USA; e Mayo Clinic Health System, Sports Medicine, La Crosse, WI, USA; f George Mason University, Kinesiology, Manassas, VA, USA; g University of the Incarnate Word, Division of Human Performance, San Antonio, TX, USA; h George Mason University, Sport, Recreation, and Tourism Management, Manassas, VA, USA

**Keywords:** Body composition, athletes, percent body fat, air displacement plethysmography

## Abstract

**Background:**

Research has shown air displacement plethysmography (ADP) to be a valid and reliable alternative to dual-energy X-ray absorptiometry (DXA) in the general population; however, its validity and reliability indicate conflicting evidence in an athletic population. The purpose of this study is to develop a novel estimation equation using body density obtained from ADP in an athletic population to assess body fat percent (BF%) more accurately.

**Methods:**

One hundred and thirty (males, *n* = 69; females, *n* = 61) National Collegiate Athletic Association Division I athletes participated in this study. Thirty athletes were randomly withheld for the hold-out sample, and the remaining 100 athletes were used in the development of the equation. Body composition was evaluated using ADP and DXA. Linear regression was used to develop a new prediction equation (Equation 1) with body density (ADP) as the independent variable and BF% (DXA) as the dependent variable. Repeated measures analysis of variance was used to identify differences between ADP–Brozek, DXA, and Equation 1. Interclass correlation coefficients (ICCs) were assessed to evaluate the reliability of the equation.

**Results:**

Significance was set to *p* < 0.05. Linear regression was conducted to create Equation 1, which explained 90.5% of the variance. ADP–Brozek reported lower BF% than Equation 1 (*p* < 0.001) in the development and hold-out samples; however, BF% from Equation 1 and DXA were not different from each other (*p* = 0.999). ICCs were strong in both samples (original sample: ICC = 0.975, *p* < 0.001; hold-out sample: ICC = 0.964, *p* < 0.001).

**Conclusion:**

The newly proposed equation may be used with ADP measurements to interpret BF% in an athletic population.

## 1. Introduction

Body composition assessment is a common practice to obtain baseline measures, monitor progress, inform training, and nutritional programming, and evaluate health status in competitive athletes. This kind of assessment can provide measures of percent body fat (BF%) and fat-free mass (FFM). Unfavorable body composition in athletes (i.e. excessively high or low body fat, insufficient FFM) has been associated with decrements in performance, as well as negative health outcomes, such as an increased risk for musculoskeletal injuries, low energy availability, and impaired immune function [1]. Therefore, it is imperative to obtain accurate measures of BF% and FFM.

There are several approaches to evaluating body composition, depending upon the available equipment and desired outcomes or level of detail regarding different tissue types. The four-compartment (4c) model is often used as a criterion method for assessing body composition [2], as it can provide information on BF%, protein content, total body water, and mineral content. However, the utility of a 4c model outside of research and clinical settings is limited due to the need for trained technicians, the cost of equipment, the time needed to conduct assessments, and data processing procedures [2]. Thus, many athletic program personnel may not view it as a feasible method to assess body composition.

Dual-energy X-ray absorptiometry (DXA) is a three-compartment (3c) model technique used to obtain BF%, bone mineral content, and lean body mass. This method has been reported to be valid in collegiate athletes (*r* = 0.87–0.94; standard error of the estimate = 2.6–2.9%) for the measurement of BF% compared to a 4c model [3]. However, the limitations associated with DXA, such as cost and the requirement for certified technicians, limit its utility as a practical option in field-based settings. Therefore, DXA may not be accessible to many intercollegiate athletic programs.

Air displacement plethysmography (ADP) is a more cost-effective alternative to DXA for body composition assessment, does not omit radiation, and does not require licensure to operate in any state. ADP provides a two-compartment (2c) model to obtain fat mass and FFM, derived from body density. This method has been shown to produce valid and reliable body composition parameters in the general population when compared to DXA [4–6]; however, conflicting evidence exists on its validity and reliability in an athletic population. Previous research has shown no difference between ADP and DXA [7,8], while other studies have reported both underestimations and overestimations of BF% [9–11]. It is possible that these discrepancies could be influenced by individual differences among the study cohorts, or the selection of the estimation equation used. While the Brozek and Siri equations were developed and shown to be valid and reliable in the general population to estimate measures of BF% from body density [4–6], they may not be applicable to individuals possessing greater proportions of FFM [10]. Previous literature has demonstrated that athletes possess more FFM than non-athletes [12] and, therefore, it is plausible that athletes may require their own unique estimation equation to estimate BF% from body density.

Due to the practicality of ADP in an athletic setting, a more accurate estimation equation for BF% may be advantageous for routine body composition assessments. This may lead to more efficient training and dietary interventions to enhance athletic health and performance, along with providing a more valid measure of changes in BF% over time. Therefore, the purpose of this study was to create a novel prediction equation for BF% in National Collegiate Athletic Association (NCAA) Division I (DI) athletes using ADP-derived parameters.

## 2. Materials and methods

### 2.1. Experimental approach to the problem

A cross-sectional approach was used to develop a novel estimation equation for determining BF% from ADP-derived parameters. Athletes arrived at the laboratory twice for the completion of a body composition assessment using ADP and a DXA scan within 48 h. Of the athletes that participated in this study (*n* = 130), 30 were randomly allocated to a hold-out sample group and were not used to develop Equation 1 and instead used to cross-validate the newly developed equation.

### 2.2. Subjects

A total of 69 male (height = 185.9 ± 9.8; body mass = 82.8 ± 12.3; 29% Black) and 61 female (height = 169.5 ± 8.3; body mass = 68.8 ± 13.5; 23% Black) NCAA DI athletes served as subjects. Athletes were participating in the following sports: basketball (males, *n* = 12; females, *n* = 12), tennis (females, *n* = 9), soccer (males, *n* = 30; females, *n* = 12), lacrosse (females, *n* = 23), and baseball (males, *n* = 32). They followed sport-specific training regimens and activities with specific neuromuscular demands. Data were collected during two separate study visits, separated by a maximum of 48 h, to obtain ADP and DXA values. Athletes were instructed to refrain from exercise, eating, and drinking for at least 2 h prior to testing. All testing was conducted in the morning to ensure the above guidelines were followed. Participants were deemed eligible if they were NCAA DI athletes, who were medically cleared for intercollegiate athletic participation by the sports medicine staff. Prior to participation in this study, athletes provided written consent. This study was approved by University Institutional Review Board (IRB #1548816–3).

### 2.3. Procedures

#### 2.3.1. Air displacement plethysmography

Upon arrival to the laboratory, height and body mass were recorded to the nearest .01 cm and .01 kg, respectively, with shoes off. A stadiometer (Detector, Webb City, MO, USA) and digital scale (Bod Pod; Cosmed, Chicago, IL, USA) were utilized. Body composition was assessed through ADP (Bod Pod body-composition system, model 2000A; Life Measurement Instruments, Concord, CA, USA) using the Brozek equation [13]. The Brozek equation was used in the present study, as it is commonly used within athletic populations [14–19]. Before each testing session, calibration procedures were conducted according to manufacturer guidelines using an empty chamber and a 49.55 L calibration cylinder. Athletes were instructed to wear unpadded compression shorts or spandex, a sports bra (female), and a swim cap with the hair tucked in. They were asked to remove all jewelry to reduce air displacement. Athletes entered the Bod Pod and sat in an upright position with hands folded on their laps. Lung gas volume was estimated using manufacturer guidelines. The Brozek equation [13] was used to estimate BF%. Following ADP assessment BF% and body density (body weight/volume) were recorded.

#### 2.3.2. Dual-energy X-ray absorptiometry

Within 48 h of ADP assessment, athletes arrived at the laboratory for DXA (Hologic, Horizon A model, Hologic Inc., Waltham, MA, USA) assessment in the morning to ensure the same guidelines for ADP testing were followed. The DXA has been shown to be a valid and reliable measure of body composition [20]. Calibration was performed in accordance with manufacturer guidelines. Athletes were instructed to wear metal-free clothing and to remove all jewelry. Athletes laid on their backs on the assessment table with their hands flat on either side of their body and feet turned inward with toes touching. They were asked to maintain this position for the duration of the scan. Athletes were scanned using the whole-body scan mode (Hologic APEX software, ver. 5.5.3.1, Bedford, MA, USA). All assessments were completed under the supervision of a trained technician. BF% was recorded following the scan.

### 2.4. Statistical analyses

Athlete descriptive characteristics were calculated as means ± standard deviation. Prior to conducting statistical analyses, 30 athletes were randomly removed from the sample and used as a hold-out to establish validity of the developed equation. Thus, 100 athletes were used in the development of a new prediction equation. Linear regression was used to produce a new prediction equation (Equation 1) to estimate BF% from body density derived from ADP. BF% obtained from DXA was used as the outcome variable and body density obtained from ADP was used as the independent variable after mean centering the variable. To mean center body density, the body density of each participant was subtracted from the average body density of the whole sample. A repeated measures analysis of variance (RMANOVA) was used to evaluate differences in BF% from the new prediction equation, Brozek equation, and DXA. If significance was observed, LSD pair-wise comparisons post-hoc analyses were conducted to identify where significance occurred. Equation 1 was applied to the hold out sample, and RMANOVA was used to determine if any differences exist between ADP-Brozek, DXA, and Equation 1. If significance was detected, LSD pairwise comparisons were used to identify where the significance occurred. To evaluate the validity of the equation, interclass correlation coefficient (ICC) was evaluated in the development sample and assessed again in the hold-out sample using BF% obtained from DXA as the criterion.

Bland–Altman and linear regressions were run to determine the systematic and proportionate agreement between BF% from Equation 1 and DXA in the development and hold-out sample. The difference between assessments was normally distributed; therefore, the assumptions of Bland–Altman plots were met. Visualizations of comparisons were created using Bland–Altman plots including 95% limits of agreement (mean bias ± (1.96 × standard deviation of differences)). Due to the potential error in interpretation of Bland–Altman plots, linear regression was used to assess the agreement between the new prediction equation and DXA [21,22]. Systematic and proportional bias was noted when 95% confidence interval of the intercept did not include “0” and “1.0,” respectively. Significance was set a priori to *p* ≤0.05. All statistical procedures were conducted in the Statistical Package for the Social Sciences (SPSS, Version 29.0; IBM Corp., Armonk, NY, USA).

## 3. Results

Athlete descriptive information is shown in Table 1. Table 1 highlights the significant differences in BF% in the development and hold-out sample, highlighting the need of creating a new estimation equation. *Equation 1* was significant (*p* < 0.001, *R*^*2*^ of 0.905).

(1) $$\mathrm{BF}\%=\;21.125-\;\left(280.823\;*\;\mathrm{mean}\;\mathrm{centered}\;\mathrm{body}\;\mathrm{density}\right)$$

**Table 1.** Equation 1 development and hold-out sample characteristics.

	Development Sample			Hold-Out Sample		
	Total	Male (*n *= 54)	Female (*n *= 46)	Total	Male (*n *= 15)	Female (*n *= 15)
Height (cm)	178.9 ± 12.3	186.7 ± 9.5	169.3 ± 7.8	176.1 ± 11.7	183.0 ± 10.3	168.9 ± 8.2
Body Mass (kg)	76.5 ± 14.2	82.6 ± 11.2	68.9 ± 14.0	75.5 ± 15.5	83.7 ± 15.2	66.9 ± 9.6
Body Density (g/cm^3^)	1.058 ± .020	1.073 ± 0.009	1.041 ± 0.015	1.057 ± 0.018	1.072 ± 0.012	1.042 ± 0.011
Percent Body Fat –- Brozek	17.7 ± 8.2*^	11.6 ± 3.8	25.0 ± 6.5	18.2 ± 7.3*^	12.3 ± 4.8	24.2 ± 4.6
Percent Body Fat – DXA	21.0 ± 6.0	16.5 ± 2.3	26.6 ± 4.3	21.4 ± 4.9	17.1 ± 2.8	25.8 ± 2.4
Percent Body Fat – Equation 1	21.0 ± 5.7	16.9 ± 2.6	26.0 ± 4.3	21.4 ± 5.0	17.3 ± 3.4	25.5 ± 3.1

Values are represented as mean±standard deviation.

*indicates significant difference when compared to DXA (*p *< 0.001).

^indicates significant difference when compared to Equation 1 (*p *< 0.001).

**Figure 1.**
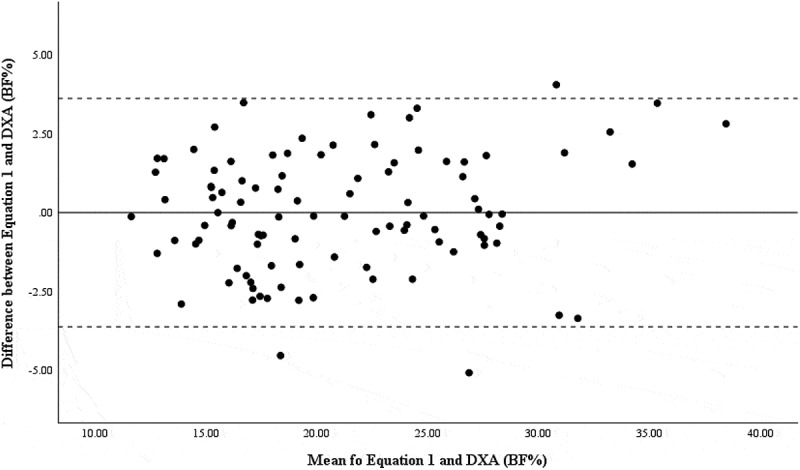
Bland–Altman plot for BF% between Equation 1 and DXA in the development sample.

**Figure 2.**
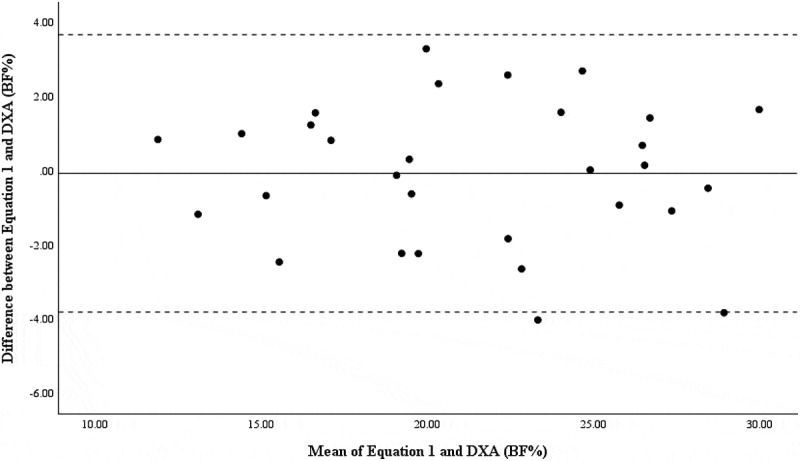
Bland–Altman plot for BF% between Equation 1 and DXA in the hold-out sample.

Results of the RMANOVA indicate BF% from Brozek was significantly lower from DXA (*p* < 0.001) and Equation 1 (*p* < 0.001) in the development sample (Table 1). Measures of BF% as assessed via DXA and Equation 1 were not different from one another (*p* = 0.999). In the hold-out sample, BF% from ADP-Brozek was significantly lower than DXA (*p* < 0.001) and Equation 1 (*p* < 0.001). BF% derived from DXA and Equation 1 was no different from one another (*p* = 0.999). Bland–Altman plots indicated good agreement between Equation 1 and DXA for the development sample (Figure 1) and the hold-out sample (Figure 2). Additionally, systemic and proportional bias was not detected by the linear regression (Table 2).

The solid line represents the mean difference in BF% between ADP and DXA, while the dashed lines represent the upper and lower 95% limits.

The solid line represents the mean difference in BF% between ADP and DXA, while the dashed lines represent the upper and lower 95% limits.

**Table 2.** Linear regression and Bland–Altman results for agreement of BF% between Equation 1 and DXA.

Sample	*R*^*2*^	Intercept (CI_95%_)	Slope (CI_95%_)	Bland–Altman Statistics	
Development	0.026	−1.088 (−2.475, 0.300)	0.052 (−.012, .115)	*t* = −1.556	*p* = 0.123
Hold-out	0.003	0.400 (−2.852, 3.653)	−0.022 (−0.170, 0.126)	*t* = 0.252	*p* = 0.762

ICCs were evaluated in the development sample and the hold-out sample of 30 participants. In both groups, the ICC was found to be significant (original sample: ICC = 0.975, *p* < 0.001; hold-out sample: ICC = 0.964, *p* < 0.001).

## 4. Discussion

The purpose of the present study was to develop a new estimation equation to assess BF% in an athletic population using ADP-derived measures of body density. Results indicate that ADP significantly underestimates BF% in athletes when compared to DXA; therefore, athletes may require an additional equation to estimate BF% more accurately. BF% predicted from Equation 1 showed good agreement in the development and hold-out as supported by the Bland–Altman analysis and linear regression statistics when compared to DXA. Further, ICCs derived from the development and hold-out sample indicated strong reliability. This study not only provides novel findings regarding athlete body composition but also improves upon the utility of ADP in athletic populations for the assessment of body fat percentage.

ADP has been considered to be accurate, indirect assessments of BF%, and operates under the assumption that fat mass and FFM compartments have a density of 0.9007 g/cm^3^ and 1.1 g/cm^3^, respectively [13]. However, variability in the fat-free compartment may be the limiting factor contributing to the questionable accuracy of 2c models in athletic populations [23]. Since athletes tend to have greater FFM than non-athletes [24], it is reasonable to assume FFM may contribute to the inaccuracy of previously developed equations, such as the Siri and Brozek equations [13,23]. The findings from the current study support the theory that the atypical body composition of athletes may lead to discrepancies between body composition assessment methods, thus warranting improvements in the utility of ADP assessment.

Findings from the present study indicate that ADP underestimates BF% in men and women athletes. Previous findings in women track and field, men and women soccer, women volleyball, women softball, men wrestling, and football athletes have reported similar underestimations of 1.5–3.7% when compared to DXA [7,25,26]. However, others have reported no differences in BF% results between DXA and ADP [27], while others have shown that ADP overestimated BF% when compared to DXA [6]. However, these discrepancies may be attributed to differing participant characteristics (i.e. ethnicity and age) compared to the current sample of athletes. For example, Ballard and colleagues and Ferri-Morales and colleagues used a sample of all Euro-American women and adolescent male athletes, respectively, while the present study included Black and Caucasian college-aged athletes.

Previous literature and the present study indicate the need for the development of a new ADP equation unique to an athletic population for evaluating body composition. In the present study, Equation 1 suggests that using the body density to estimate BF% can explain 90.5% of the variance in BF% from DXA. Thus, it is reasonable to suggest that the equation has a high predictive value [28]. The Bland–Altman plots demonstrate high agreeability between Equation 1 and DXA, and proportional bias was not observed in both the development and hold-out samples [22]. This indicates Equation 1 could be used to provide a more accurate measure of BF% in athletes utilizing ADP assessments.

This is the first study in which an equation was developed that estimates BF% from ADP-derived body density within an athletic population. The strong correlation between BF% values derived from DXA and Equation 1 lends to the utility of this equation for measuring BF%. However, the current study is not without limitations. DXA was used as the criterion for BF% rather than a 4c model as we did not have the ability to assess for total body water; however, the DXA has been shown to be a valid and reliable measure in athletic populations [2,3]. Although athletes were provided with guidelines around eating and drinking prior to ADP testing, hydration status and total body water were not assessed. Additionally, ADP and DXA assessments were performed on different days due to laboratory constraints; however, all measurements were taken within 48 h of each other and done at the same time of day in the morning. While the present study had a large sample size (development sample, *n* = 100; hold-out sample, *n* = 30), continued investigation into this equation is warranted to establish further reliability and validity across additional athletic populations.

Results from the present study indicate that ADP alone yields different BF% results from DXA, which supported development of an estimation equation to predict BF% in an athletic population when using ADP-derived measures of body density. The novel equation accurately and precisely estimated BF% when using body density obtained from ADP, compared to DXA. Further, no significant differences between Equation 1 and DXA were observed. Due to constraints surrounding DXA, this novel equation may be advantageous when evaluating BF% in an athletic population. This equation can assist in obtaining more accurate measures of BF% compared to ADP alone. Further, it can better inform training and nutritional programming, as well as prevent unwanted gains and losses in fat mass and FFM, respectively.

Practitioners may wish to consider applying Equation 1 to their athletes when using ADP for body composition assessment. It is possible Equation 1 from the present study may provide more accurate body composition measurements in comparison to the standard ADP equations. This equation is easy to use in other athletic populations by subtracting 1.058 (mean body density from the present study) from the body density obtained with ADP. Next, that number would be substituted in the equation for *mean centered body density*. For example, if an athlete’s body density is 1.049, the practitioner would take the following steps to use Equation 1:

Step 1: Find *mean centered body density*: 1.058–1.049 = 0.009

Step 2: Put *mean centered body density* into *Equation 1*:

$$\;\;\;\;\;\;\;\;\;\;\;\;\;\;\;\;\;\;\;\;\;\;\;\;\;\;\;\;\;\;\;\;\;\;\;\;\;\;\;\;\;\;\;\;\;\;\;\;\;\;\;\;\;\;\;\;\;\;\;\;\;\;\;\;\;\;\;\;\;\;\;\;\;\;\;\;\;\;\;\;\;\;\;\;\;\;\;\;\mathrm{BF}\%=\;21.125-\;\left(280.823*0.009\right)$$

Step 3: Solve for BF%:BF%= 21.125 − 2.527

BF%= 18.598%
